# Lessons learned from five patients of persistent Mullerian duct syndrome: A case series

**DOI:** 10.1016/j.ijscr.2022.107459

**Published:** 2022-07-27

**Authors:** Shehryar Ahmed Khan Niazi, Muhammad Umer Mukhtar, Rameez Hassan, Qasim Mehmood

**Affiliations:** a Laparoscopic Surgeon and Surgical Oncologist, District Headquarter Hospital, Bhakkar, Pakistan; b King Edward Medical University, Pakistan

**Keywords:** Persistent Mullerian duct syndrome, Transverse testicular ectopia, Undescended testes, Mullerian malignancy

## Abstract

**Introduction:**

Persistent Mullerian duct syndrome (PMDS) is a genetic disorder characterized by the persistence of Mullerian structures of fallopian tubes, uterus, and upper two-thirds of the vagina in a normal XY male. It is a rare genetic disorder that has been reported less than two hundred times. More rarely it may be seen in association with transverse testicular ectopia (TTE).

**Presentation of cases:**

Four patients presented with swelling in the inguinal region and undescended testes. Pre-op ultrasound was done on three of these patients and it showed a hernia with testes inside the hernial sac. Hernia surgery was planned for these patients. One patient presented with a complaint of bilateral cryptorchidism that prompted laparoscopic exploration. In all five patients, on surgical exploration, Mullerian derivatives i.e., fallopian tubes, uterus, and vagina were found in the abdomen. Hernia surgery was done and Mullerian structures were excised. For undescended testes, patients had orchiopexy or orchidectomy depending on their respective age group.

**Discussion:**

PMDS is caused by failure of production of Mullerian inhibiting substance. Mullerian structures other than causing inguinal hernia are also at risk of malignant transformation, which is the most important significance of this condition. In light of the risk of malignant transformation, Mullerian structures must be excised.

**Conclusion:**

To prevent the risk of malignant transformation in PMDS, the Mullerian structures must be excised. If PMDS is associated with TTE, orchiopexy must be done for pediatric patients and orchidectomy for adult patients.

## Highlights

• Persistent Mullerian duct syndrome (PMDS) is a genetic disorder characterized by the persistence of Mullerian structures.
• PMDS is associated with a risk of malignancy in Mullerian structures if not treated.
• PMDS is rare and the risk of malignancy stresses wider recognition of this disorder.
• Excision of Mullerian derivatives eliminates the risk of malignancy and is the mainstay of treatment for PMDS.

## 1 Introduction

Persistent Mullerian duct syndrome (PMDS) is a rare form of male pseudohermaphroditism characterized by a lack of regression of Mullerian duct derivatives of fallopian tubes, uterus, cervix, and upper two-third vagina in a phenotypically and genotypically normal male. It is an extremely rare disorder that has been described less than two hundred times in literature. The disorder is familial with an autosomal recessive pattern of inheritance and is characterized by mutations in genes related to the Mullerian inhibiting substance (MIS), a hormone responsible for the regression of Mullerian ducts in males. The persistent structures in the abdomen prevent normal descent of testes into the scrotum and result in the most common presentation of undescended testis (UDT) on one side and inguinal hernia on the contralateral side. In most cases, the diagnosis is made incidentally during surgery done for the UDT or hernia, although radiologic techniques can enable preoperative diagnosis [1], [2], [3]. Here, we discuss five cases of PMDS in compliance with the PROCESS 2020 guidelines and later address the diagnostic challenges, treatment options, and other dilemmas that the surgeon may face in the management of this rare disease [4].

## 2 Case presentation

### 2.1 First patient

**Fig. 1:**
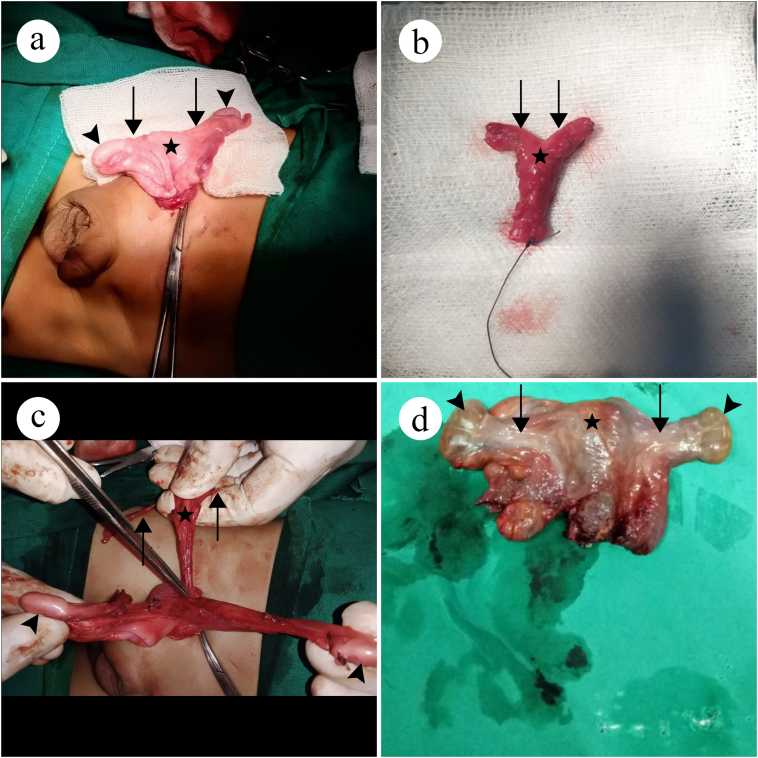
Mullerian structures. *Stars* indicate uteruses, *arrows* indicate fallopian tubes and *arrowheads* indicate testes.

A two-year-old male presented with swelling in the left inguinal region. The patient had had hernia surgery for another left-sided hernia a month ago. The surgery was followed by immediate hernia recurrence, which the surgeon of the prior surgery had attributed to edema. The patient was referred for a redo surgery to the surgeon at a community hospital. On exam, the patients swelling was 1.5 × 2 cm, ovoid, reducible, and nontender. External genitalia were normal but there were bilateral undescended testes. Hernia surgery was planned. During surgical exploration, cecum, appendix, and terminal ileum were found in the hernial sac. In addition, bilateral fallopian tubes, uterus, and part of vagina were also found in the hernial sac, with both testes attached to the fallopian tubes at their fimbrial ends (Fig. 1a) (Video 1). Thus, PMDS was diagnosed. Bilateral orchiopexy and resection of Mullerian structures was done by a specialist surgeon. To correct the hernia, hernioplasty was done. Post op course was unremarkable.

### 2.2 Second patient

A two-year-old male presented with swelling in the right inguinoscrotal region for the past 1 month. On exam, the swelling was 1.5 × 1.5 cm, round, reducible, and nontender. There was a scar on the left side which had occurred due to a hernia surgery that the patient had a year ago. The patient had normal male external genitalia. Both testes were absent from the scrotum. Ultrasound was performed and it revealed the presence of a loop on intestine and both testes in the hernial sac. On surgical exploration, in addition to the loop of intestine and both testes, Mullerian structures of fallopian tubes, uterus, and part of vagina were also found. PMDS with TTE was diagnosed. As the patient was only 2 years old and there was a chance of fertility, bilateral orchiopexy was done. Mullerian structures were excised (Fig. 1b) and hernioplasty was done. Post-op was uneventful.

### 2.3 Third patient

**Fig. 2:**
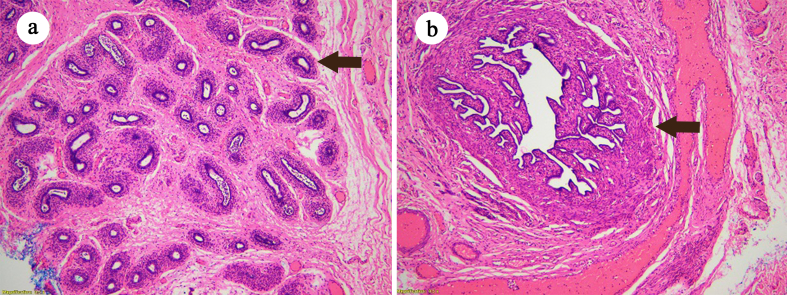
Histopathology. a: Viable testicular tissue, *arrowhead* indicates a seminiferous tubule. b: *Arrow* indicates resected fallopian tube.

A five-year-old male presented with a history of bilateral undescended testes. The patient had seen a pediatric surgeon in the past for his cryptorchidism. On laparoscopic exploration, the pediatric surgeon had confused the undescended testes in the abdomen of the patient as ovarian tissue. Due to inexperience, the pediatric surgeon referred the patient to the author team. The author team took a biopsy of the suspected testes. Histopathology confirmed the presence of viable testicular tissue. (Fig. 2a) In light of this, bilateral orchiopexy was planned. On surgical exploration, along with confirmation of the bilateral undescended testes, Mullerian structures of fallopian tubes, uterus, and vagina were also found (Figs. 1c and 2b) (Video 2). Bilateral orchiopexy was done and Mullerian structures were excised. Post op course was unremarkable.

### 2.4 Fourth patient

A thirty-two-year-old male presented with a swelling in the left inguinoscrotal region for the last six months. The swelling was ovoid, painless, and reducible. The right hemiscrotum was empty while a testis was present on the left side. External genitalia and secondary sex characters were normal. Ultrasonography showed a hernial sac on the left side containing a loop of bowel and omentum. Both testes were also reported to be on the left side. Thus, a diagnosis of inguinal hernia with TTE was made and surgery was planned. On surgical exploration, both testes and Mullerian structures were also found in the hernial sac. (Fig. 1d) Bilateral orchidectomy and hernioplasty were done. Mullerian structures were excised. Postoperatively, the patient was started on testosterone replacement therapy.

### 2.5 Fifth patient

A thirty-year-old male presented with a swelling in the right inguinoscrotal region that had been gradually enlarging for the past several years. On exam, the swelling was globular and reducible. Cough impulse was positive. External genitalia and secondary sex characters were normal. The right testis was present in the right hemiscrotum with the left hemiscrotum being empty. Ultrasonography showed a hernial sac containing a loop of intestine and the presence of both testes in the right inguinal canal. Thus, a diagnosis of inguinal hernia with TTE was made. During surgery, along with a loop of intestine and both testes, Mullerian structures were also found in the hernial sac. The hernia was reduced and hernioplasty was done. Bilateral orchidectomy was performed and Mullerian structures were excised. Postoperatively, the patient was started on testosterone replacement therapy.

## 3 Discussion

PMDS is a rarely reported autosomal recessive disorder. In normal male development, during the seventh week, the Sertoli cells of the fetal testes produce MIS, which leads to the regression of the Mullerian structures. Genetic mutations leading to failure of production or release of MIS, defective MIS, or defective MIS receptor all lead to PMDS. In contrast, as the physiology of testosterone and dihydrotestosterone is unaffected in patients of PMDS, they have normal development of male internal and external genitalia and thus are phenotypically male. Due to its genetic basis, the disorder is associated with consanguinity [5]. Four out of five of our cases were the progeny of consanguineous marriages.

PMDS has been classified into three types based on anatomy: Type I which is called the “male form” is the most common and is characterized by the presence of testes alongside the Mullerian structures in the hernial sac. In some cases of the “male form”, the Mullerian derivatives can be drawn into the hernial sac, which in turn can pull the contralateral testis into the hernial sac. Type II which is the least common type is called the “female form” and is characterized by the presence of testes inside the abdomen attached to the fimbrial end of the fallopian tubes, mimicking the position of the ovaries in a normal female. Type III is PMDS associated with TTE, a condition in which both testes are present on the same side, with either or both being in the abdomen, inguinal canal, scrotum, or elsewhere [3]. Among our cases, the first patient had PMDS type II, the second and third patients had PMDS type I and the fourth and fifth were of PMDS type III or PMDS with TTE.

PMDS is not only rare but is difficult to diagnose as well. The most common presentation is UDT on one side and inguinal hernia on the other. In most cases, the absent testes are found inside the hernial sac. Diagnostic techniques of ultrasound, CT, and MRI can visualize the Mullerian structures and thus can help make a preoperative diagnosis; but only if the surgeon's suspicion prompts the use of these techniques. Otherwise, as in the majority of cases, diagnosis is made unexpectedly during a surgical correction for the UDT or hernia. It is worthwhile to note that radiological techniques are subjective and may result in false negatives. Thus, laparoscopy remains the gold standard for diagnosis of PMDS [1].

The main points of consideration in the management of a patient of PMDS are the preservation of fertility and the risk of malignancy in Mullerian derivatives. The undescended testes are exposed to an unfavorable temperature of the abdomen, which adversely affects the function of the temperature-sensitive Sertoli cells and results in infertility. Dysplasia in the testes and maldevelopment of the epididymis may also be causes of infertility. By rule, patients of PMDS are infertile [3]. Both of our adult patients were infertile. As far as malignancy in a patient of PMDS is concerned, it may arise either in the testes or the Mullerian structures. Possible testicular malignancies include embryonal carcinoma, seminoma, yolk sac tumor, or teratoma. The risk of malignancy in testes (5–18 %) is far more than the risk of malignancy in Mullerian structures (3.1–8.4 %) [6], [7], [8]. Studies that have followed patients have reported the occurrence of endocervical adenocarcinoma and uterine adenosarcoma among malignancies arising in Mullerian structures [3].

In our cases, patients one and two had had surgery for hernia and in the third case, the patient had had exploration done for cryptorchidism during which the diagnosis of PMDS had escaped the prior surgeons that were unfamiliar with this entity. It was only after exploration done by surgeons familiar with the disease that the disease was identified and resection made possible. Thus, the risk of missing a diagnosis of PMDS and resultantly leaving a child at risk of malignant transformation, heavily stresses the need for more widespread recognition of this disease.

The treatment of PMDS has been subject to debate. As stated before, the main concerns are preservation of fertility and prevention of malignancy. One school of thought recommends orchiopexy for the UDT and advises against the removal of Mullerian structures. This disapproval of resection of Mullerian structures is due to the difficulty in dissection, risk of damage to vas deferentes, and the presumed low risk of malignancy in Mullerian structures [5]. The second school of thought recommends orchiopexy and removal of all Mullerian structures. The third school of thought recommends the removal of both Mullerian structures and cryptorchid testes to eliminate all risk of malignancy. In our opinion, the second manner of treatment is preferable as it attempts to preserve fertility (via orchiopexy), eliminates the risk of Mullerian malignancy, and spares the patient from lifelong follow-up (via Mullerian resection) [9]. Laparoscopic approach enables easy resection of Mullerian structures and orchiopexy with minimal trauma. The ideal time for orchiopexy is between the age of one to two years.

## 4 Conclusion

PMDS is a rare genetic disorder. A high level of suspicion is required in cases of unilateral hernia and contralateral undescended testes to diagnose this condition preoperatively. Diagnosis can be confirmed by radiologic investigations and laparoscopy. The authors recommend laparoscopic resection of Mullerian remnants with orchiopexy or orchidectomy as a treatment for this disease.

The following are the supplementary data related to this article.

Video S1 Persistent Mullerian Duct Syndrome: Intraoperative Discovery. Video S1

Video S2 Practical Considerations During Resection. Video S2

## Funding

This study had no funding sources.

## Ethical approval

Ethical approval was taken from Chairperson of Department of Surgery, DHQ Hospital, Bhakkar, Pakistan.

## Consent

Informed written consent was taken for the patients or their parents for publication of the cases and the relevant media.

## Author contribution

**Shehryar Ahmed Khan Niazi**: Conceptualization, Methodology, Investigation, Resources, Supervision, Project administration. **Muhammad Umer Mukhtar**: Conceptualization, Software, Data curation, Writing- Original draft preparation, Writing - Review & Editing, Visualization. **Rameez Khan**: Methodology, Investigation, Resources, Supervision. **Qasim Mehmood**: Conceptualization, Validation.

## Registration of research studies

1. Name of the registry: UMIN-CTR
2. Unique identifying number or registration ID: R000054477UMIN000047784
3. Hyperlink to your specific registration (must be publicly accessible and will be checked): https://center6.umin.ac.jp/cgi-open-bin/ctr_e/ctr_view.cgi?recptno=R000054477

## Guarantor

Shehryar Ahmed Khan Niazi.

## Declaration of competing interest

None to declare.
